# Capturing synchronization with complexity measure of ordinal pattern transition network constructed by crossplot

**DOI:** 10.1098/rsos.221067

**Published:** 2023-06-28

**Authors:** Xiaobi Chen, Guanghua Xu, Bo He, Sicong Zhang, Zijvn Su, Yaguang Jia, Xun Zhang, Zhe Zhao

**Affiliations:** ^1^ School of Mechanical Engineering, Xi'an Jiaotong University, Xi'an 710049, People's Republic of China; ^2^ State Key Laboratory for Manufacturing Systems Engineering, Xi'an Jiaotong University, Xi'an 710049, People's Republic of China; ^3^ School of Microelectronics, Xi'an Jiaotong University, Xi'an 710049, People's Republic of China; ^4^ The First Affiliated Hospital of Xi'an Jiaotong University, Xi'an, People's Republic of China; ^5^ School of Materials, Sun Yat-sen University, Shenzhen 518107, People's Republic of China

**Keywords:** synchronization, crossplot, ordinal pattern transition network, transition entropy

## Abstract

To evaluate the synchronization of bivariate time series has been a hot topic, and a number of measures have been proposed. In this work, by introducing the ordinal pattern transition network into the crossplot, a new method for measuring the synchronization of bivariate time series is proposed. After the crossplot been partitioned and coded, the coded partitions are defined as network nodes and a directed weighted network is constructed based on the temporal adjacency of the nodes. The crossplot transition entropy of the network is proposed as an indicator of the synchronization between two time series. To test the characteristics and performance of the method, it is used to analyse the unidirectional coupled Lorentz model and compared it with existing methods. The results showed the new method had the advantages of easy parameter setting, efficiency, robustness, good consistency and suitability for short time series. Finally, electroencephalogram (EEG) data from auditory-evoked potential EEG-biometric dataset are investigated, and some useful and interesting results are obtained.

## 1. Introduction

Since Huygens first described the phenomenon of synchronization when observing the coupling of a nearby pendulum in 1665 [1], the synchronization between dynamical systems has been one of the main research directions of nonlinear dynamics. Synchronization and related phenomena have been observed not only in engineering and economic systems, but also in biological and physiological systems [2–6]. However, despite the fact that synchronization is a ubiquitous phenomenon, the detection and quantification of synchronization can be a non-trivial task, as the phenomenon can manifest itself in many different ways. As a result, various concepts and methods for synchronization detection have been proposed for this purpose. In order to ensure that the analysis is controllable, the system with monotonic behaviour in one direction is often deliberately selected for synchronization analysis. Usually, the maximum Lyapunov exponent is used as an indicator of the presence of synchronization in the driven system, with a negative value indicating that synchronization has been achieved [7]. Previous analyses have shown that the maximum Lyapunov exponent has a rather monotonic dependence on the coupling strength, which can be used as a first control parameter for the discrimination of different degrees of synchronization [8–10]. Roughly speaking, the existing synchronization analysis methods can be divided into three main groups: linear methods, nonlinear methods and spectral analysis methods [11]. Using three different coupled model systems to create a ‘controlled’ environment, the literature [12] compared six analysis methods belonging to these three groups in terms of their ability to distinguish between different levels of coupling and their robustness to noise. Although the linear approach is computationally simple and physically meaningful, it is not sufficient to deal with nonlinear dependencies. In most cases, nonlinear methods quantify synchronization by making certain assumptions rather than directly estimating a nonlinear function. In recent years, these have become mainstream analysis methods and are widely used to detect synchronization. One of the existing nonlinear methods is based on the information theory, such as mutual information (MI) [13,14], permutation mutual information (PMI) [15] and cross-sample entropy (CSE) [16,17]. These methods have certain practical value in practical applications. However, these methods have requirements on the length and stationarity of the time series when analysing. Note that most of the data collected in practice are limited in length and non-stationary, which limits the application of the above methods. For the case where stationarity holds only for short time series, Hempel *et al*. [18] proposed a permutation-based measure, inner composition alignment (IOTA), which facilitates the study of interactions between very short time series. Although the IOTA method has the advantage of being able to identify the degree of automatic adjustment between time series and can be applied to very short time series, the analysis results of slightly longer time series are easily affected by extreme values. To compensate for the shortcomings of the IOTA, inspired by the permutation entropy and IOTA, Shi *et al*. [19] proposed the cross-permutation entropy (CPE) by reconstructing the phase space of the time series and integrating the idea of IOTA. By analysing artificial series and stock markets, CPE can find the relationship between the two synchronous time series, which has the advantages of sample, stable and efficient. Phase synchronization, as another nonlinear method, can detect phase synchronization even if there is no correlation between the amplitudes of two coupled chaotic signals [20]. Several phase synchronization-based indicators have been proposed, such as the phase locking value (PLV) [21], phase lag index (PLI) [22] and weighted phase lag index (wPLI) [23]. The PLV is a temporal synchonization measure frequently applied in electroencephalography (EEG). Both PLI and wPLI are the improvement of PLV mainly to eliminate the influence of the volume conductor effect, in which the wPLI considers the amplitude and phase factors in the calculation. In addition, as a very promising analysis method, recurrence plot has also received increasing attention in synchronization analysis. The advantage of recurrence plot is that it is suitable for non-stationary and very short time series. Marwan *et al*. proposed a cross-recurrence plot (CRP) to quantify the synchronization of two time series of the same type [24]. Marwan *et al*. further proposed that the joint recurrence plot (JRP) can graphically describe the simultaneous recurrences in two or more dynamical systems [25]. The existing research results show that JRP is a useful candidate method for discovering generalized synchronization [26,27].

With the in-depth study and application of statistical analysis and complex network theory, crossplot [28–30] and ordinal pattern transition networks (OPTN) [31–34] are emerging as major tools for nonlinear time series interrelationship analysis and are finding meaningful applications in the analysis of experimental data in a variety of research fields. Crossplot is a graphical representation of time series in the Cartesian plane. The scatter point in the plot is represented by the paired points of two continuous time series. For experimental data, the underlying dynamics are usually not completely known. Crossplot can accurately and quickly visualize the relationship between two continuous time series. This makes it easier to uncover potentially important relationships and answer questions about how one variable is influenced by another. However, a crossplot that ignores the temporal order of the scatter points cannot avoid the issue of data point overlap when there are slightly more time series data points. This will cause the performance of the crossplot to degrade [35]. Originally proposed by Small [36], the OPTN is being established in the hope that network measures will provide new information about the dynamics that generate the time series. To generate OPTN, ordinal patterns [37] after time series symbolization are used as nodes of the network, and directed edges are determined based on the temporal adjacency of the ordinal patterns, resulting in a weighted and directed graph. Once the networks have been created, various network parameters can be calculated to provide a better understanding of the underlying dynamics of the time series. Studies have shown that [38,39] OPTN is an effective means of quantifying deterministic dynamical properties and detecting changes in the behaviour of complex chaotic data and biological systems. Ordinal patterns are chosen for their simplicity and sensitivity to temporal causality, as well as their robustness to noise, by discarding amplitude level information in the symbolization process. OPTN can not only analyse univariate time series, but also be extended by Subramaniyam *et al*. to reliably identify the direction of interaction and the coupling delay in the analysis of multivariate time series [40]. Crossplot and OPTN have each found some interesting applications in scientific research and engineering, but so far, they have not yet been fused and integrated into time series synchronization analysis based on their respective merits.

In this paper, by introducing OPTN into the crossplot, a new method for measuring the synchronization of bivariate time series is proposed. To achieve a tight integration of two methods, the acquisition of ordered patterns is achieved by partitioning and coding the crossplot. By comparing the effects of grid square box partition and phase radial partition on unidirectional coupled Lorenz model, the rationality of the phase radial partition scheme is determined. The partitioned crossplot is coded and uses codes as network nodes. A directed weighted network is constructed based on the temporal adjacency of network node. The weights of the network edges are the frequency of transitions between nodes. The transition entropy of the OPTN is used as an evaluation indicator of the coupling strength. Compared with PMI, CPE, PLV, PLI and JRP methods, the proposed method analysis result of the unidirectional coupled Lorenz model under different coupling strengths shows that the new method combines the advantages of crossplot and OPTN. On the one hand, the symbolization of crossplot reduces the influence of noise. On the other hand, the temporal adjacency of ordinal patterns solves the problem of overlapping data point in the crossplot. In addition, the measures based on OPTN have clear physical meaning, high efficiency and are easy to quantify. To verify the validity of the method for real data, EEG data from auditory-evoked potential EEG-biometric dataset are analysed using the proposed method.

This paper is structured as follows: in §2, we introduce the method employed in our work. The construction and coding process of crossplot is introduced, followed by a detailed description of the generation process of crossplot OPTN and the concept of crossplot transition entropy (CPTE). In addition, PMI, CPE, PLV, PLI and JRP methods are briefly introduced. In §3, the determination process of the relevant parameter is introduced and the unidirectional coupled Lorenz system is analysed. The results are also compared with five other methods for the coupled model. In addition, the effect of Gaussian white noise on the analysis results is also considered. Next, real auditory-evoked potential EEG-biometric dataset is analysed. The discussion and conclusion are given in §4 and §5, respectively.

## 2. Methods

This section first describes the crossplot ordinal pattern transition network (COPTN) proposed in this paper, and then briefly introduces five other common methods for analysing time series synchronization, namely PMI, CPE, PLV, PLI and joint-order recurrence plot (JORP).

### 2.1. Crossplot ordinal pattern transition network

**Figure 1.**
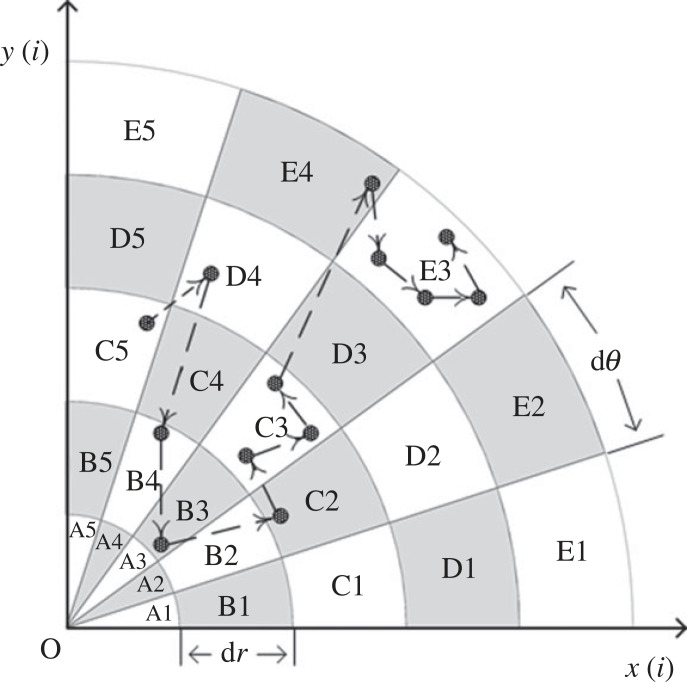
Schematic diagram of crossplot partitioning and encoding.

**Figure 2.**
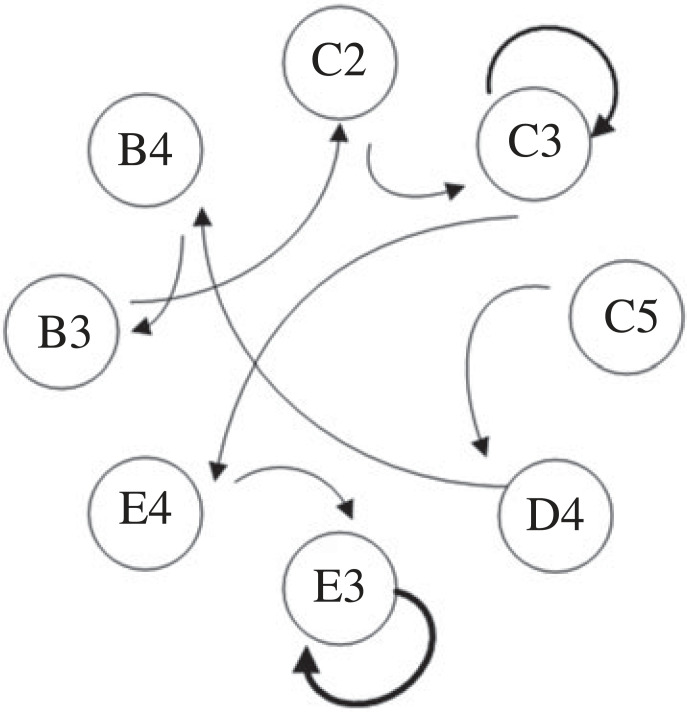
The schematic diagram of the directed weighted network constructed from figure 1.

Here we define the process for mapping a crossplot into OPTN. This process basically includes two parts. The first part introduces the construction process of crossplot and its partition coding method. The second part is to build a directed weighted network according to the temporal adjacency relationship of each partition, using the coded partitions as network nodes. To simplify the analysis, the time series is converted to a time series that does not contain negative values, so that all the scatter points fall in the first quadrant of the Cartesian coordinates. The realization process is as follows:

(1) For two continuous time series $x_{i}^{\prime }$ and $y_{i}^{\prime }(i=1\cdots N)$ with a synchronous relationship, calculating the respective minimum values $x_{\min}^{\prime }$ and $y_{\min}^{\prime }$, and the minimum values $x_{\min}^{\prime }$ and $y_{\min}^{\prime }$ are subtracted from time series $x_{i}^{\prime }$ and $y_{i}^{\prime }(i=1\cdots N)$ to obtain two time series *x_i_* and $y_{i}(i=1\cdots N)$ with no negative values.
(2) The crossplot is constructed using the coordinates $\left\{(x_{i},y_{i}),(i=1\cdots N)\right\}$. Where *x_i_* is the abscissa and *y_i_* is the ordinate. All scatter points of the constructed crossplot lie in the first quadrant of the Cartesian coordinates.
(3) The constructed crossplot is partitioned and coded. Partitioning uses the scheme of integrating the phase and amplitude information contained in the scatter points. The partitioning and coding process is shown in figure 1, first dividing the quadrant evenly into *m* sectors, with each sector coded counterclockwise by 1, 2, 3,…, followed by making *n* concentric circles with the origin as the centre and *dr* as the ruler. Coding is done in radial order using capital letters A, B, C…. After the above two steps, the crossplot is divided into sector ring subintervals along the radial direction. For the convenience of analysis, *m* is taken as a fixed value of 9 in here and subsequent analysis, that is, each sector angle *dθ* is 10°. The value of *dr* is determined by the distribution of the scatter points. The value of *n* can be obtained by rounding upwards the quotient of the distance from the farthest scatter point to the origin and the ruler *dr*:
  2.1 $$n=\left\lceil \frac{r_{\max}}{dr}\right\rceil ,$$
  where *r*_max_ is the maximum distance from the scatter point to the origin.
(4) For the coded crossplot, the code of each sector ring subinterval is used as a network node, and a directed weighted network is constructed according to the temporal adjacency relationship between nodes, with the number of transitions as the weight of the network. Taking figure 1 as an example to illustrate the construction process of the directed weighted network. The temporal adjacency relationships of coded scatter points are: C5 → D4 → B4 → B3 → C2 → C3 → C3 → C3 → E4 → E3 → E3 → E3 → E3. The schematic diagram of the constructed directed weighted network is shown in figure 2, where the thickness of the line in the graph is proportional to the frequence of transitions. Observing figure 2, it can be seen that nodes E3 and C3 form self-loops, which means that the same state transition occurs at nodes C3 and E3.
(5) The constructed network needs to be reflected by appropriate quantitative indicators. To better disclose the difference transition behaviour between ordinal patterns, a discrete probability set of transition behaviours is constructed based on the constructed directed weighted network. This is illustrated by the set of discrete probabilities of the transition behaviour of the weighted directed network in figure 2. The conversion frequencies between the nodes in figure 2 are 1, 1, 1, 1, 1, 2, 1, 1, 3. Because the total number of transition is 12, the probability set $p_{S_{A}\to S_{B}}$ (*S_A_*, *S_B_* ∈ {*A*1, *A*2, … , *E*4, *E*5}) is {1/12, 1/12, 1/12, 1/12, 1/12, 1/6, 1/12, 1/12, 1/4}. Based on discrete probability set $p_{S_{A}\to S_{B}}$, the regularity of the ordinal pattern transition properties can be quantified by Shannon entropy *H*_tr_, the crossplot transition entropy (CPTE), which is
  2.2 $$H_{\mathrm{tr}}=-\sum \;p_{S_{A}\to S_{B}}\log_{2}(p_{S_{A}\to S_{B}}),$$
(6) where *S_A_*, *S_B_* ∈ {*A*1, *A*2, … , *E*4, *E*5}, we use log_2_ and hence the units of *H*_tr_ are bits. Here, *H*_tr_ is suggested as an indicator of the synchronization of the two time series.

### 2.2. Permutation mutual information

Mutual information represents the intersection of two time series, and it is very effective to measure the linear and nonlinear dependencies of two time series [41]. The larger the value of mutual information, the stronger the information interaction between the two time series. It is often used to study the synchronization between time series [14,42]. However, the estimation of probability density functions of time series has been a challenge and has given rise to a variety of different approaches [43–46]. In order to avoid the complicated calculation process of probability distribution function, Zhao *et al*. [15] proposed PMI based on the idea of permutation entropy, which simplified the calculation process of mutual information.

For two equal-length continuous time series {*x*(*t*)} and {*y*(*t*)}*t* = {1, 2, … , *N*}, the phase space reconstruction is performed by the time delay parameter *τ* and the embedding dimension *d*. The state vectors obtained from the two time series are $X_{t}=[x_{t},x_{t+\tau },x_{t+2\tau },\cdot \cdot \cdot x_{t+(d-1)\tau }]$ and $Y_{t}=[y_{t},y_{t+\tau },y_{t+2\tau },\cdot \cdot \cdot y_{t+(d-1)\tau }]$, and the permutation vectors $\pi_{x_{i}}$ and $\pi_{y_{i}}$ i∈{1,2,⋅⋅⋅N−(d−1)τ} are calculated in ascending order, respectively. The respective permutation entropy *P*(*x*) and *P*(*y*) were calculated according to the Bandt and Pompe method [37]. The joint probability distribution of the two permutation vectors is

2.3 $$p(\pi_{x_{i}},\pi_{y_{i}})=\frac{\#\{X_{i},Y_{i}|(X_{i},Y_{i})\;\mathrm{has}\;\;\mathrm{pattern}(\pi_{x_{i}},\pi_{y_{i}})\}}{N-(d-1)\tau }.$$

Based on $p(\pi_{x_{i}},\pi_{y_{i}})$, calculate the joint permutation entropy of two time series

2.4 $$P(x,y)=-\sum_{i=1}^{N-(d-1)\tau }\;p(\pi_{x_{i}},\pi_{y_{i}})\log_{2}p(\pi_{x_{i}},\pi_{y_{i}}).$$

According to the definition of mutual information, the PMI is calculated as follows:

2.5 PMI(x,y)=P(x)+P(y)−P(x,y).

### 2.3. Cross-permutation entropy

Inspired by permutation entropy and IOTA method, CPE was proposed when Shi *et al*. studied the relationship between financial time series [19]. It uses an IOTA method to detect the cross-correlation between two synchronization time series, which has the advantages of sample, stable and efficient.

(1) For two equal length continuous time series {*x*(*t*)} and {*y*(*t*)} t={1,2,⋅⋅⋅,N}, the phase space reconstruction is performed using the delay parameter *τ* and the embedding dimension *d* to calculate the state vectors $X_{t}=[x_{t},x_{t+\tau },x_{t+2\tau },\cdot \cdot \cdot x_{t+(d-1)\tau }]$ and $Y_{t}=[y_{t},y_{t+\tau },y_{t+2\tau },\cdot \cdot \cdot y_{t+(d-1)\tau }]$, t∈{1,2,⋅⋅⋅N−(d−1)τ}.
(2) Perform a non-decreasing permutation on the vector *X_t_*, and denote its permutation position by *π_X_*. Taking the permutation position *π_X_* as the standard, rearrange the vector *Y_t_*, and the result is recorded as *G_t_* = *Y_t_*(*π_x_*). If *X_t_* and *Y_t_* are fully synchronized state vectors, *G_t_* will be a monotonically increasing sequence, otherwise not.
(3) Using the idea of IOTA, monotonicity is quantified by counting the number of intersections of the horizontal line drawn by each data point of *G_t_* and *G_t_* itself. The number of intersections of the *k*th state vector is calculated using the following equation:
  2.6 $$k_{t}=\sum_{i=1}^{d-2}\sum_{\;j=i+1}^{d-1}\Theta [(G_{t}(j+1)-G_{t}(i))(G_{t}(i)-G_{t}(j))],$$
  where Θ [*x*] is Heaviside function:
  2.7 $$\Theta [x]=\{\begin{array}{c} 1,x>0\\ 0,x\leq 0 \end{array}.$$
(4) According to this method, all state vectors of the time series are traversed, and the number of intersections of each state vector can be expressed as a unique integer *z*, *z* ∈ [0, *R*], *R* = (*d* − 1)(*d* − 2)/2 is the maximum possible number of intersections. For all the *R* + 1 possible integer $z_{i},\;i=0,1,\cdot \cdot \cdot ,R$ of intersection points *k_t_* in each state vectors, its relative frequencies can be obtained by
  2.8 $$p(z_{i})=\frac{\#\{k_{t}|k_{t}=z_{i}\}}{N-(d-1)\tau },$$
  where 1 ≤ *t* ≤ *N* − (*d* − 1)*τ*, 0 ≤ *i* ≤ *R*, # is the number of elements in it. Then, after obtaining the probability distribution set $P=\{p(z_{i}),i=1,\cdot \cdot \cdot ,R\}$, CPE can be defined as
  2.9 $$H_{x\to y}(d,\tau )=-\sum_{i=0}^{R}\;p(z_{i})\log_{2}p(z_{i}).$$

### 2.4. Phase locking value

As one of the most used synchronization measures, PLV describes the stability of the phase difference between two time series in a subsequent time sample window [47]. If *ϕ*_1_ and *ϕ*_2_ are the phases of two time series, and Δ*ϕ* is the phase difference, PLV can be defined as the locking of the phases associated with each signal, such as

2.10 $$|n\phi_{1}-m\phi_{2}|=\mathrm{const}.$$

Usually, the two signals analysed are from the same type of signal, and the phase locking ratio is taken as *n* : *m* = 1 : 1. The instantaneous phase *ϕ*_1_ and *ϕ*_2_ of the two time series can be obtained by applying the Hilbert transform to them. The phase difference Δ*ϕ* is calculated after obtaining the instantaneous phases *ϕ*_1_ and *ϕ*_2_. Then, the PLV can be calculated according to the following equation:

2.11 $$\mathrm{PLV}=\left\langle e^{i\Delta \phi }\right\rangle =\left|\frac{1}{N}\sum_{k=0}^{N-1}e^{i\Delta \phi (t_{k})}\right|.$$

Where, *t_k_* are discrete time-steps and *N* is the number of samples. PLV takes values in the space of [0, 1], where 1 indicates perfect phase synchronization and 0 indicates a lack of synchronization.

### 2.5. Phase lag index

As a measure of the asymmetry in the distribution of the instantaneous phase difference between two time series, PLI was proposed to quantify phase synchronization in order to reduce the effect of volume conduction problem [22]. Just as PLV, PLI uses the phase difference in equation (2.11) to find phase consistency and it was defined by

2.12 $$\mathrm{PLI}=\left|\frac{1}{N}\sum_{k=0}^{N-1}\mathrm{sign}(\sin(\Delta \phi (t_{k})))\right|,$$

where ‘sign’ is the signum function. PLI ranges between 0 and 1, and values close to 1 indicate stronger non-zero locking.

### 2.6. Joint-order pattern recurrence plot

The joint recurrence plot (JRP) graphically describes the joint probability of simultaneous recurrence in the phase space of two or more dynamical systems [25]. The JRP of two powertrain systems can be defined as

2.13 $$\mathrm{JRP}=\mathrm{Heaviside}(\varepsilon^{x}-\|x_{i}-x_{j}\|)\cdot \mathrm{Heaviside}(\varepsilon^{y}-\|y_{i}-y_{j}\|).$$

Here, *i*, *j* = 1, … , *N*, ε*^x^* and ε*^y^* are two thresholds for the two systems. Heaviside is the Heaviside(*t*) function that yields 0 if *t* < 0 or 1 otherwise. Existing research indicates that the JRP are more appropriate for studying two interacting systems and may be useful for detecting generalized synchronization [26,27]. Usually, the values of the thresholds ε*^x^* and ε*^y^* have a significant impact on the analysis results. Many scholars had contributed their efforts to alleviate the problem of threshold selection. By studying the recurrence characteristics of the order patterns in the symbolic sequence to remove the influence of the threshold, Chen *et al*. proposed the order pattern recurrence plot (OPRP) [48]. Following the Bandt and Pompe method, a continuous time series is encoded into a symbolic sequence with 'Taken' phase space embedding to obtain order patterns [37]. Then, we replace the distance-based recurrence in JRP with the order pattern-based recurrence to construct JORP. The JORP is a graphical representation of the order pattern recurrence matrix of *x* and *y* when recurrence occurs simultaneously, which can be obtained as

2.14 $${\mathrm{JORP}}_{i,j}=\{\begin{array}{cc} 1 & \mathrm{if}\;\pi_{i^{\prime }}^{x}=\pi_{{\;j}^{\prime }}^{x}\;\mathrm{and}\;\pi_{i^{\prime }}^{y}=\pi_{{\;j}^{\prime }}^{y}\\ 0 & \mathrm{otherwise}.\; \end{array}$$

Here, $\pi_{i^{\prime }}^{x}$, $\pi_{{\;j}^{\prime }}^{x}$ and $\pi_{i^{\prime }}^{y}$, $\pi_{{\;j}^{\prime }}^{y}$ (*i′*, *j′* = 1, … , *N*_op_) are order patterns of time series *x* and *y* at time *i* and *j*. *N*_op_ is the order pattern symbolic sequence length.

Referring to the research results of the literature [27], the recurrence rate (RR) of JORP is adopted as the indicator to measure the synchronization of two time series and can be obtained as follows:

2.15 $$\mathrm{RR}=\frac{1}{N_{\mathrm{op}}^{2}}\sum_{i,j=1}^{N_{\mathrm{op}}}{\mathrm{JORP}}_{i,j}.$$

## 3. Analysis and results

In this section, to demonstrate that the proposed method can be used to characterize synchronization, we apply two types of time series: the unidirectional coupled Lorenz model and the auditory evoked potential EEG-biometric dataset to examine the effectiveness and compare the results with other methods.

### 3.1. Analysis of coupled dynamic model

#### 3.1.1. Unidirectional coupled Lorenz model

The coupling dynamic model adopts the unidirectional coupled Lorenz model, which is often used in synchronous analysis [1,12], because the maximum Lyapunov exponent of its response system shows more complex fluctuations than other systems [12].

The unidirectional coupled Lorenz model is identified by

3.1 $$\begin{array}{c} {\dot{x}}_{1}=10(x_{2}-x_{1})\\ {\dot{x}}_{2}=x_{1}(28-x_{3})-x_{2}\\ {\dot{x}}_{3}=x_{1}x_{2}-\frac{8x_{3}}{3} \end{array},$$

as the driving system and

**Figure 3.**
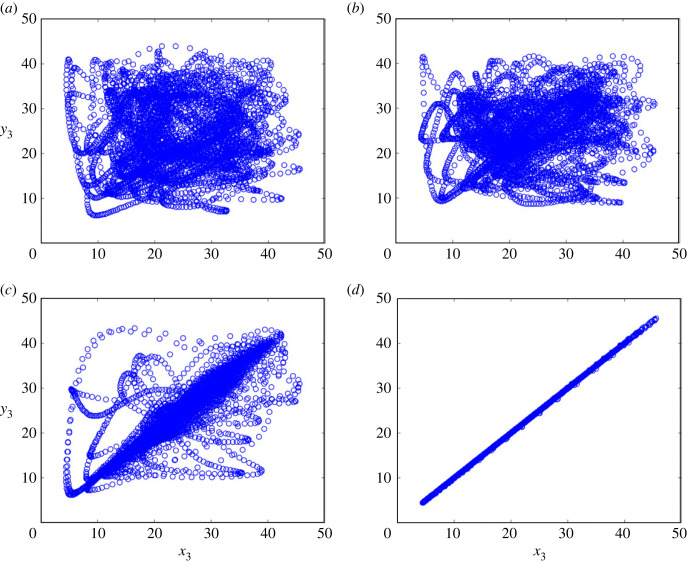
Crossplots constructed by coupled time series *x*_3_ and *y*_3_ at different coupling strengths. (*a*) *C* = 0.3, (*b*) *C* = 0.5, (*c*) *C* = 1.2, (*d*) and *C* = 1.6.

3.2 $$\begin{array}{c} {\dot{y}}_{1}=10(y_{2}-y_{1})\\ {\dot{y}}_{2}=y_{1}(28.001-y_{3})-y_{2}\\ {\dot{y}}_{3}=y_{1}y_{2}-\frac{8y_{3}}{3}+C(x_{3}-y_{3}) \end{array},$$

as the response system, where *C* is the strength of the coupling and is varied from 0 to 2 in steps of 0.1. The unidirectional coupled Lorenz is generated using the 4th-order Runge–Kutta integration method with integration step sizes of 0.01 and 50 000 iterations. The initial value is chosen randomly between 0 and 1. The first 30 000 transient data points are discarded and time series consisting of remaining 20 000 data points are analysed. Figure 3 shows the crossplots constructed by the coupled time series *x*_3_ and *y*_3_ when the coupling strength is 0.3, 0.5, 1.2 and 1.6, respectively. It can be observed from figure 3 that with the increase of coupling strength, the distribution of scatter points tends to be closer to the 45° line of the crossplot.

#### 3.1.2. Rationality of partition scheme

The proposed partition scheme for the crossplot is strongly anisotropic and inhomogeneous. The area of each sector ring subinterval after partition is not uneven. The further away from the point of origin, the larger the area will be. This partition scheme is intuitively inconsistent with the homogeneous partition scheme that is commonly adopted. In order to explain the rationality of the partition scheme adopted, two partition schemes are used to compare the CPTE of crossplot constructed by coupled time series *x*_3_ and *y*_3_ at different coupling strengths. One partition scheme is using a grid of square boxes, and the other is the proposed partition scheme. The length of continuous time series *x*_3_ and *y*_3_ is 20 000 samples. The coupling strength *C* is 0.3, 0.5, 0.9, 1.2 and 1.6, respectively. The sliding time window with a window width of 2000 samples is used to segment the time series in steps of 500 samples. Also, in order to observe the influence of ruler size on the analysis results, the ruler is incremented in 1 from 1 to 30.

**Figure 4.**
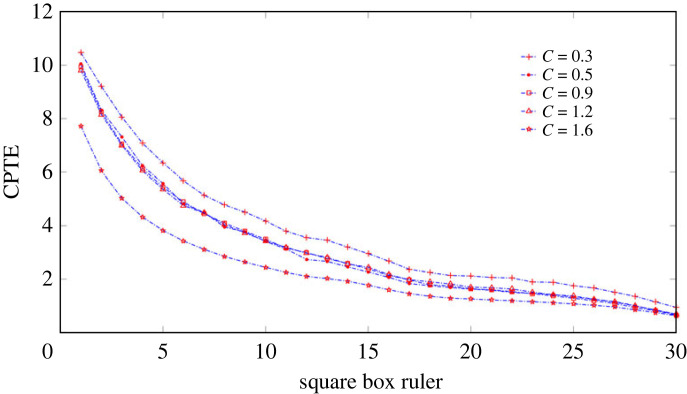
The average value of CPTE for 30 times of repeated calculation under different rulers when the square box was used to partition crossplot, with the square side length as the ruler.

**Figure 5.**
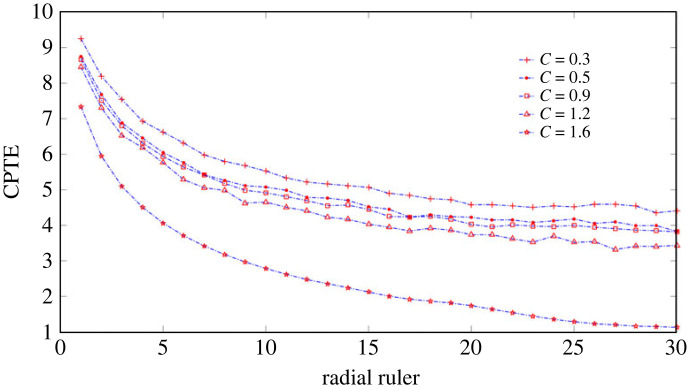
The average value of CPTE calculated 30 times repeatedly under different rulers when the proposed partition scheme was used to partition the crossplot, with the radial segment length as the ruler.

At a certain coupling strength, crossplot, builted by consecutive time series *x*_3_ and *y*_3_ within the same sliding window, is partitioned and constructed OPTN. The CPTE obtained from each crossplot is averaged as a measure of this coupling strength. When grid partition is adopted, the side length of the square is used as the ruler. When the ruler changes from small to large, the analysis results of two partition schemes under different coupling strengths are shown in figures 4 and 5, respectively. The results of figures 4 and 5 are the average of the results of 30 repeated calculations. Comparing figures 4 and 5, it can be found that using grid square boxes to partition the crossplot cannot effectively distinguish coupled time series with coupling strengths of 0.3, 0.5 and 0.9 at all ruler. But, the proposed partition scheme can make an effective distinguish. To explore the reasons for this, one may be due to the non-uniform nature of the distribution of the time series data, with more opportunities for small values to occur and fewer opportunities for large values to occur. Two, it may be that the scattered phase angle has a prominence effect on the results, but instead the effect of the phase angle is ignored with the grid square box partition. Therefore, the proposed partitioning scheme is more reasonable. It is also noticeable that CPTE decreases as the ruler increases, with the rate of decrease being fast when the ruler is small and slowing down as the ruler increases. The value of the ruler has no influence on the identification of the coupling state, but too small a ruler increases the computational load and too large a ruler ignores some information. In general, the ruler value is chosen to correspond to the transition region where the decrease rate of the CPTE value tends to level off. Thus, in the following analysis, the ruler value is chosen to be 10.

### 3.2. Consistency of CPTE and synchronization

**Figure 6.**
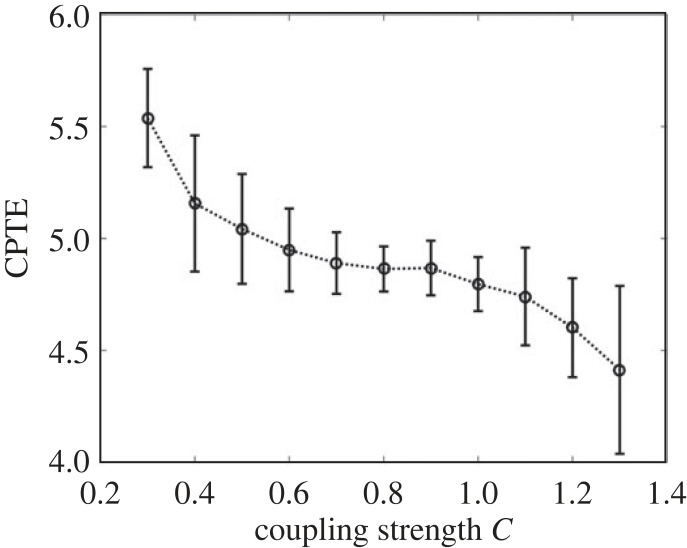
The CPTE and its standard deviation calculated with the radial scale *dr* = 10 and the coupling strength *C* within the range from 0.3 to 1.3.

To illustrate the relationship between CPTE and coupling strength, the coupled time series *x*_3_ and *y*_3_ with coupling strength *C* in the range from 0.3 to 1.3 are analysed using the sliding time window. The sliding window width is 2000 samples and the step size is 500 samples. The CPTE at each coupling strength is repeatedly calculated 30 times. The average CPTE of all sliding windows is taken as the measured value under a certain coupling strength. The results obtained are shown in figure 6.

To quantify the consistency between CPTE and coupling strength, the concept of monotonicity degree in the literature [12] is introduced.

3.3 $$M_{\mathrm{CPTE}}=\frac{2}{r(r-1)}\sum_{i=1}^{r-1}\sum_{\;j=i+1}^{r}\mathrm{sign}({\mathrm{CPTE}}_{j}-{\mathrm{CPTE}}_{i}),$$

where *r* is the number of coupling strength *C*, *i* < *j*. It attains the value *M*_CPTE_ = 1 for a strictly monotonically increasing sequence (CPTE_1_,…, CPTE*_r_*), while it renders −1 for a monotonically decreasing sequence.

Calculating the monotonicity degree *M*_CPTE_ of the sequence of CPTE values in figure 6, and the result is −0.9636. This shows that the proposed measures CPTE have a strong negative correlation with the coupling strength *C*.

#### 3.2.1. Effect of sliding window width on analysis results

**Figure 7.**
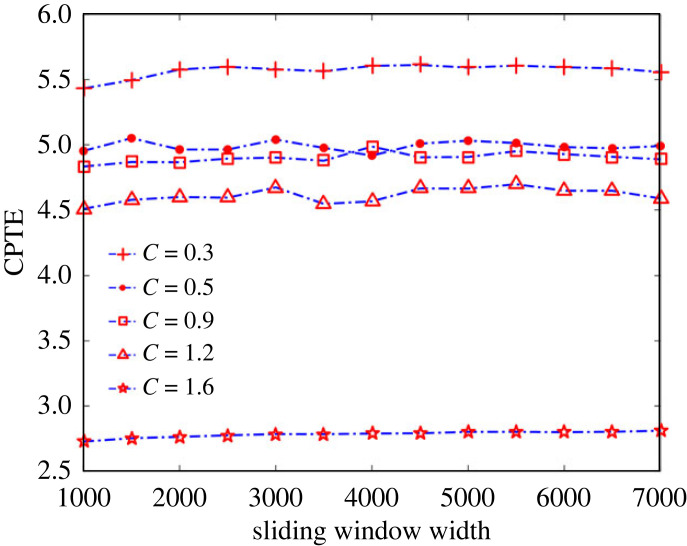
The average value of CPTE calculated 30 times repeatedly under different sliding window width with the radial ruler *dr* = 10. The coupling strength *C* are 0.3, 0.5, 0.9, 1.2, and 1.6, respectively.

The width of the sliding time window in the above analysis is fixed at 2000 samples. To examine whether the size of the sliding window width has an effect on the CPTE, the coupled time series *x*_3_ and *y*_3_ are analysed using sliding time windows under different window widths in a step size of 500 samples. The sliding window width varies in increments of 500 samples over a range of 1000–7000 samples. Time series with coupling strengths of 0.3, 0.5, 0.9, 1.2 and 1.6 are selected for analysis, respectively. The average CPTE of all sliding windows is taken as the measured value under a certain window width. Each coupling strength is repeatedly calculated 30 times. The results in figure 7 show that the CPTE is almost independent of the sliding window width after the window width is greater than 2000 samples. This also shows that the proposed method is suitable for the synchronization analysis of short coupled time series.

#### 3.2.2. Comparison of synchronization analysis results

**Figure 8.**
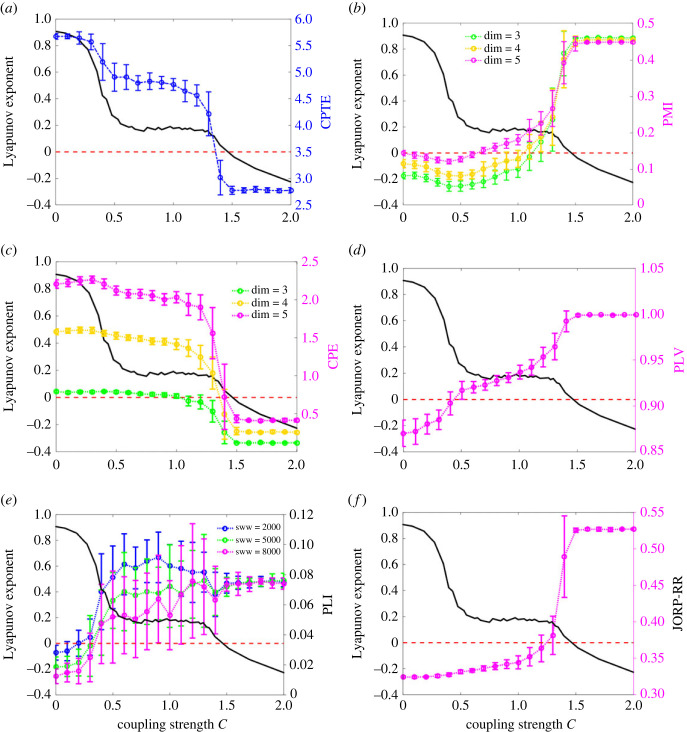
At different coupling strength *C*, the synchronization analysis results (right ordinate value) of the unidirectional coupled Lorenz model using three methods are compared with the maximum Lyapunov exponent (black solid line, left ordinate value) of the response system. The sliding time window width is 2000 samples, and the step size is 500 samples. The red dotted line is the horizontal zero line of Lyapunov exponent. (*a*) When *dr* is 10, the mean and standard deviation of the CPTE with the coupling strength *C* (30 repeated calculations). (*b*) When the delay time is 12 and the embedded dimensions are 3, 4 and 5, respectively, the mean and standard deviation of the PMI with the coupling strength *C* (30 repeated calculations). (*c*) When the delay time is 12 and the embedded dimensions are 3, 4 and 5, respectively, the mean and standard deviation of the CPE with the coupling strength *C* (30 repeated calculations). (*d*) The mean and standard deviation of the PLV with the coupling strength *C* (30 repeated calculations). (*e*) The mean and standard deviation of the PLI with the coupling strength *C* (30 repeated calculations) when the sliding window width (sww) was taken as 2000, 5000 and 8000 samples, respectively. (*f*) The RR mean and standard deviation of the JORP with the coupling strength *C* (30 repeated calculations), dim = 5 and *τ* = 12.

Taking the maximum Lyapunov exponent of the response system as the theoretical reference, the analysis results of the proposed method and five other methods for the same coupled time series are compared. The average of all sliding time windows is used as the measured value. The parameters of each method are set as follows. For CPTE, the radial ruler *dr* is 10. For PMI, CPE and JORP, according to the literature [1], the delay time *τ* is set to 12. The embedding dimension *dim* can only be a maximum of 5 due to the limitation of the window width. In order to provide more comprehensive information, the embedding dimensions for PMI and CPE are taken as 3, 4 and 5, respectively. For JORP, the results calculated under different embedding dimensions vary greatly, and it is not possible to show detailed information under all three embedding dimensions at the same time, so its embedding dimension is 5. At each coupling strengths *C*, 30 repeated calculations are performed for all methods. Figure 8 illustrates the analysis results. All results are displayed in double ordinates to better show the difference between the analytical results of each method and the theoretical values. The left ordinate value is the theoretical value of the maximum Lyapunov exponent, and the right ordinate value represents the measured value of each method. The complete synchronization status of the model is detected by all methods when the Lyapunov exponent of the response system is less than zero. However, for regions where the maximum Lyapunov exponent is greater than 0, different methods exhibit different detection capabilities. The analysis results of CPTE, PLV and JORP have good consistency with the changes of coupling strength, and all show changes in the same or opposite direction. The analysis results of PMI and CPE show opposite evaluation results when the coupling strength is less than 0.5, indicating that the evaluation results of PMI and CPE are unreliable when the coupling strength is less than 0.5. When the sliding window width is 2000 sample points, the least ideal result is PLI, and the results in the coupling strength range of 0.5–1.5 are greater than those in the complete synchronized state. To determine whether PLI is affected by the sliding window width, 5000 and 8000 samples were also taken as sliding window width for analysis. It was found that as the window width increased, the results of PLI analysis tended to be ideal. This suggests that PLI has a requirement for sliding window width and is not applicable to short time sequences. Therefore, only the CPTE, PLV and JORP methods are used in the next analysis oriented towards the real dataset.

The computational efficiency of the three methods is also compared. The time required to complete a unidirectional coupled Lorentz model coupling analysis using the sliding time window parameters described above is used as a comparison. The configuration of the computer used is: CPU-Intel(R) Core(TM) i5-3320 M CPU @ 2.6 GHz, RAM-8.00G, Operating systems-Windows 7 64bit. Mean and standard deviation were calculated for the time of 30 replicate treatments, and the results are shown in table 1. Table 1 shows that the three methods CPTE, PLV and PLI take less computing time to complete an analysis and are more efficient. The computational efficiency of CPTE is lower than that of PLV and PLI.

**Table 1.** The time required to complete a unidirectional coupled Lorenz model coupling analysis using sliding time window (time/seconds).

mean ± s.d.	dim = 3	dim = 4	dim = 5
PMI	2.4463 ± 0.0552	2.6001 ± 0.0741	2.7068 ± 0.0827
CPE	0.3353 ± 0.0178	0.3626 ± 0.0130	0.3978 ± 0.0354
JORP	2.8461 ± 0.0898	2.8931 ± 0.0686	2.9696 ± 0.0951
CPTE	0.1130 ± 0.0048		
PLV	0.0124 ± 0.0025		
PLI	0.0108 ± 0.0023		

The results are the mean and standard deviation of the 30 measurements.

#### 3.2.3. Effect of noise on CPTE

**Figure 9.**
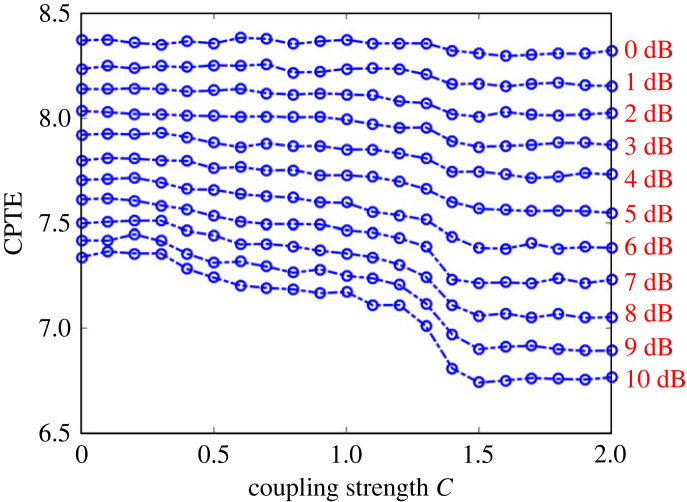
Variation of CPTE with coupling strength *C* with different levels of Gaussian white noise added to the response system. The SNR decreases from 10 dB to 0 dB in descending order from the bottom to the top. Each point shows the average across 30 iterations.

Noise can blur the synchronization between two time series. Here, the effect of different noise levels on the synchronization of the unidirectional coupled Lorenz model is analysed. Gaussian white noise was added to the response system of the unidirectional coupled Lorenz model, with the signal to noise ratio (SNR) increasing from 10 dB to 0 dB in steps of −1 dB. The values of the radial ruler *dr* and sliding time window parameters are the same as in the previous section. Figure 9 clearly shows how the CPTE varies as a function of the coupling strength *C* for different noise levels. Under different coupling strength *C*, the values of CPTE increases with the increase of noise level, which indicates that noise will affect the analysis results of CPTE. At SNR greater than 1 db, the CPTE values still can reflect the effect of different coupling strengths *C* on synchronization, and at SNR less than 1 db, CPTE failed as an indicator of synchronization. The results of the above analysis show that the method proposed in this paper is robust to noise.

### 3.3. Analysis of auditory-evoked EEG signals

To demonstrate the application of the proposed method in practical data, the signals of auditory-evoked potential EEG-biometric dataset [49] are analysed to investigate the inter-channel interaction under various types of auditory stimulation conditions. The dataset can be downloaded from the website: https://www.physionet.org/content/auditory-eeg/1.0.0/. It included over 240 two-minute EEG recordings obtained from 20 volunteers. Each subject's data included resting state EEG signals with eyes open and closed and six experimental EEG signals from auditory stimuli. Three of the auditory stimulus experiments were in-ear auditory stimulus and the other three were bone-conduction auditory stimulus. The three stimuli for each case are a native song, a non-native song and neutral music.

The equipment used for the experiments was the OpenBCI Ganglion Board and the supporting software was OpenBCI GUI v. 5.0.3. It consisted of four Gold Cup electrodes that were placed in positions T7, F8, Cz and P4 after application of Ten20 conductive paste to the scalp according to the 10/10 international EEG system, which was selected according to these publications [50,51]. In addition, two electrodes were placed in the left and right ear as the reference electrode and the ground electrode, respectively. Once all the electrodes were installed, a simple calibration was performed to ensure that everything was working correctly. The calibration consisted of checking the connection of the electrodes by measuring the skin impedance. Two minutes of EEG data were segmented from the raw data, with the lowest possible noise, i.e. when the subject was moving and blinking less.

The EEG signals contained in each subject after segmented are as follows:

(1) 2 min of resting state, eyes open for three sessions;
(2) 2 min of resting state, eyes closed for three sessions;
(3) 2 min of listening to a song in their native language using in-ear headphones;
(4) 2 min of listening to a song in a non-native language using in-ear headphones;
(5) 2 min of listening to neutral music using in-ear headphones;
(6) 2 min of listening to a song in their native language using bone-conducting headphones;
(7) 2 min of listening to a song in a non-native language using bone-conducting headphones;
(8) 2 min of listening to neutral music using bone-conducting headphones.

Some subjects had less than 2 min of data after segmentation. Therefore, the length of data selected for analysis was 20 000 samples per subject. There were three sessions of data for each subject with eyes open and eyes closed in the resting state. In the analysis, the data from the first session were taken uniformly for eyes open and eyes closed in the resting state to facilitate the analysis. For the segmented data, the trend terms of the EEG signals were first removed using the singular value decomposition (SVD) method. The detrended signals were then filtered using a harmonic wavelet with a phase-locked function in the frequency range of 4–32 Hz to obtain the EEG signals to be analysed. Four EEG electrodes T7, F8, Cz and P4 were combined in pairs to form a total of six paired electrodes: T7-F8, T7-Cz, T7-P4, F8-Cz, F8-P4 and Cz-P4. For enhanced data analysis, the paired EEG signals were split into five epochs, each containing 4000 paired samples. The sliding time window method is used to analyse each epoch. The window length of the sliding window was 2000 samples, and the step size was 500 samples. The CPTE of each sliding window was calculated, and the average value of all sliding time windows were taken as measured value of this epoch. When calculating CPTE, the radial ruler *dr* was 2. The CPTE values of five epochs were averaged as synchronization measure for each subject for the paired electrode. For the convenience of expression, different auditory evoked environments are abbreviated. Resetting state with eyes open is abbreviated as Reop, resetting state with eyes closed is abbreviated as Recl, listening to native song using in ear headphones is abbreviated as Inna, listening to non-native song using in ear headphones is abbreviated as Innn, and listening to neutral music using in ear headphones is abbreviated as Inne. Listening to native song using bone conducting headphones is abbreviated as Bona, listening to non-native song using bone conducting headphones is abbreviated as Bonn and listening to neutral music using bone conducting headphones is abbreviated as Bone.

**Figure 10.**
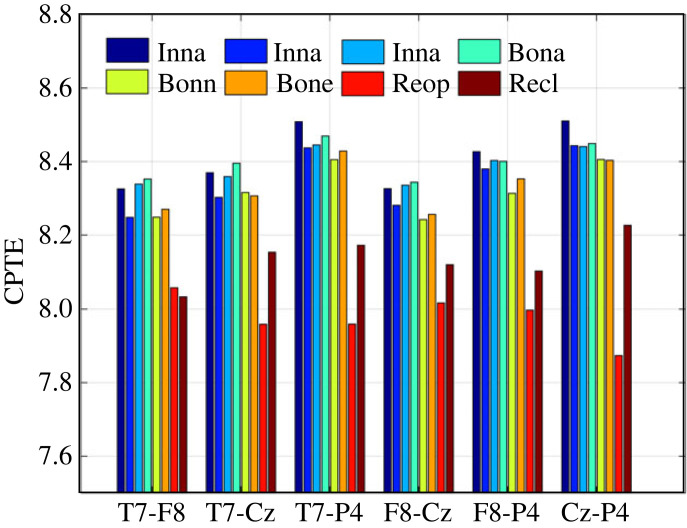
Under different auditory evoked conditions, the changes of synchronization between electrode pairs expressed by CPTE. Each CPTE shows the average across 20 subjects.

Figure 10 shows the change in synchronization between electrode pairs expressed as CPTE under different auditory stimulus environments, with the value of each bar being the average of 20 subjects. The CPTE in the resting state between each electrode pair was significantly smaller than auditory stimulus state, and in particular the CPTE in the resting state with eyes open was the smallest (T7-F8 is not obvious). This implies that the highest synchronization between the four electrodes was observed in the eyes open relaxed state and decreased with the intervention of auditory stimulus. It was also observed that the CPTE was greater in the case of listening to native song than in the case of listening to non-native song and neutral music for both in-ear and bone-conduction auditory stimulation. With inner ear headphones, the CPTE values of the P4 electrode with other electrodes are highest when listening to native songs.

To further confirm whether synchronization between electrode pairs was significantly different across auditory stimulation, the results were statistically analysed using multi-paired sample Friedman non-parametric test with the null hypothesis was (H0): there was no significant difference in the CPTE between electrode pairs under different auditory stimulus environments. The significance level was set at *p* = 0.05, and statistical analysis was performed on IBM spss25.0. The asymptotic significance of the CPTE multi-paired sample Friedman non-parametric test for electrode pairs T7-F8, T7-Cz, T7-P4, F8-Cz, F8-P4 and Cz-P4 in different auditory stimulus environments were 0.000, 0.000, 0.000, 0.001, 0.000 and 0.000, respectively. Friedman's non-parametric tests were also carried out on the analytical results of PLV and JORP. The asymptotic significance of the PLV for electrode pairs T7-F8, T7-Cz, T7-P4, F8-Cz, F8-P4 and Cz-P4 in different auditory stimulus environments were 0.336, 0.000, 0.911, 0.000, 0.045 and 0.003. The asymptotic significance of the JORP for electrode pairs T7-F8, T7-Cz, T7-P4, F8-Cz, F8-P4 and Cz-P4 in different auditory stimulus environments were 0.000, 0.158, 0.000, 0.540, 0.000 and 0.001. It can be observed that there were significant differences in all six electrode pairs under different auditory stimulus environments when CPTE method was used for analysis. The test results were not significant when using PLV analysis for electrode pairs T7-F8 and T7-P4 and when using JORP analysis for electrode pairs T7-Cz and F8-Cz in different auditory stimulus environments. To present more detailed information, there methods pairwise comparison results of each electrode pair under different auditory stimulus environments are listed in tables 2–4. When pairwise comparison, the null hypothesis is that the synchronization of each electrode pair in deferent auditory stimulus environments have the same distribution. The significance level is *p* = 0.05, and the Bonferroni correction has adjusted the significance values for multiple tests. For the convenience of display and analysis, only the adjusted significance values with significant differences for each electrode pair under two different auditory stimulus environments are presented. Observing the pairwise comparison results in tables 2–4, it can be seen that the significant changes in synchronization between electrode pairs mainly exist between resting state and auditory stimulus state, and the synchronization between electrode pairs under different auditory stimulus is not significant. The PLV method did not detect significant changes in synchronization on electrode pairs T7-F8 and T7-P4. JORP also did not detect significant changes in synchronization on electrode pairs T7-Cz and F8-Cz. Further, it can be found that JORP can only detect the synchronization of electrode pairs between eye open resting and auditory stimulus state, but not between eye closed resting and auditory stimulus state. The reason for the above phenomenon is that PLV only uses the phase information of time series, while JORP only uses the amplitude interdependence information of state space vector. Owing to the comprehensive consideration of the amplitude and phase information in the two time series, the proposed CPTE can detect more synchronization changes. Careful analysis of the pairwise comparison test results in table 2 can find more interesting results. The synchronization between P4 electrode and the other three electrodes showed more significant changes between resting state and auditory stimulation state. It suggests that the synchronization between electrode P4 and other electrodes will be changed during auditory stimulus. The number of significant changes in the synchronization of electrode pairs caused by bone conduction auditory stimulus was less than that of in-ear auditory stimulus, indicating that bone-conduction auditory stimulus had less influence on EEG under the same auditory stimulus. At the same time, it can also be found that whether using bone-conduction headphones or in-ear headphones, the number of significant changes in electrode pair synchronization when listening to native songs is more than listening to other types. This means that native songs can make significant changes in brain state. These conclusions are not observed in the pairwise test results of PLV and JORP.

**Table 2.** The pairwise comparison adjusted significance between the CPTE of electrode pairs under two different auditory stimulus environments.

adjusted significance	T7-F8	T7-Cz	T7-P4	F8-Cz	F8-P4	Cz-P4
Reop-Inna		0.001	0.000	0.014	0.000	0.000
Reop-Innn		0.044	0.002		0.017	0.000
Reop-Inne	0.035	0.000	0.000	0.028	0.017	0.000
Reop-Bona	0.035	0.000	0.000		0.028	0.000
Reop-Bonn		0.005	0.001			0.001
Reop-Bone			0.001			0.000
Recl-Inna	0.022		0.000	0.035	0.000	0.000
Recl-Innn					0.017	
Recl-Inne	0.008				0.017	
Recl-Bona	0.008	0.000			0.028	

The significance level is *p* = 0.05, and the Bonferroni correction has adjusted the significance values for multiple tests.

**Table 3.** The pairwise comparison adjusted significance between the PLV of electrode pairs under two different auditory stimulus environments.

adjusted significance	T7-F8	T7-Cz	T7-P4	F8-Cz	F8-P4	Cz-P4
Reop-Inna		0.022		0.000	0.014	
Reop-Innn		0.003		0.000		
Reop-Inne				0.000		
Reop-Bona				0.001		
Reop-Bonn		0.002		0.000		
Reop-Bone		0.004		0.001		
Recl-Inna				0.003		
Recl-Innn		0.035		0.028		0.022
Recl-Inne				0.004		
Recl-Bonn		0.022		0.035		
Recl-Bone		0.044				0.007

The significance level is *p* = 0.05, and the Bonferroni correction has adjusted the significance values for multiple tests.

**Table 4.** The pairwise comparison adjusted significance between the JORP-RR (*dim* = 5 and *τ* = 5) of electrode pairs under two different auditory stimulus environments.

adjusted significance	T7-F8	T7-Cz	T7-P4	F8-Cz	F8-P4	Cz-P4
Reop-Inna	0.000		0.000		0.000	0.011
Reop-Innn	0.005		0.000		0.000	0.008
Reop-Inne	0.001		0.004		0.002	
Reop-Bona			0.000		0.002	0.001
Reop-Bonn	0.000		0.000		0.000	0.001
Reop-Bone	0.001		0.000		0.000	0.028

The significance level is *p* = 0.05, and the Bonferroni correction has adjusted the significance values for multiple tests.

## 4. Discussion

The aim of the study is to develop a new method for measuring the synchronization of bivariate time series to achieve an expansion of the existing synchronization measures. To this end, the OPTN with clear physical meaning and easy to quantify is introduced into the crossplot that can intuitively measure the correlation between two time series. The OPTN network building method based on nodes temporal adjacency relationship ideally solves the problem of data points overlap inherent in the crossplot. It also proposes the transition entropy of OPTN as the indicator of the synchronization of two time series.

To achieve a perfect fusion of both methods, the crossplot is partitioned and coded. For the partition scheme, instead of the commonly used grid square box uniform partition scheme, a phase radial partition scheme was used based on the distribution characteristics of the scatter points [52]. In order to verify the feasibility of the proposed partition scheme, the unidirectional coupled Lorenz model with multiple coupling strengths is partitioned using two partition schemes, respectively, and the transition entropy of the constructed OPTN are calculated. The comparative results of the transition entropy obtained using two different partition schemes at different rulers confirm the rationality of the partition scheme used. This also confirms that the scatter distribution characteristics and crossplot phase angles are important factors that should be taken into account when determining the partition scheme.

It is an unavoidable problem that the proposed indicator has the ability to reflect different degrees of coupling between two time series. For this reason, taking monotonicity degree as the evaluation criterion, we calculated the monotonicity degree of CPTE sequence obtained by two coupled time series under the coupling strength increasing gradually. The results of the analysis show that the monotonicity degree of the CPTE sequence corresponding to the incremental coupling strength is −0.9363. This proves that the proposed indicator is in good agreement with the coupling strength. Considering the possible influence of sliding window width on the analysis results, CPTEs of multiple coupling strengths between time series with different window widths are calculated. The analysis results show that when the window width is greater than 2000 samples, CPTE is almost not affected by the window width. On the other hand, it also shows that the proposed method is very suitable for the short time series. Compared with the existing PMI, CPE, PLI and JORP, the state evaluation of coupling degree of the proposed method is almost independent of parameter settings and has the highest computational efficiency. In addition, by adding different intensities of Gaussian white noise one can find that it is relatively immune to additive noise. For real data, the pairwise comparison of CPTE of each electrode pair in different auditory stimulus environments showed that the effects of bone-conduction auditory stimulus and in-ear auditory stimulus on EEG were reflected in the significant changes in the synchronization of electrode pairs relative to the resting state.

The above analysis clearly demonstrates that the proposed method shows several advantages:

(1) This method requires only one parameter to be determined in use, and the value of the parameter has little or no influence on the evaluation of the synchronization state.
(2) CPTE is not affected by the length of the analysis window and requires only a small number of time series to obtain stable results, making it ideal for simultaneous analysis of short time series.
(3) Direct partitioning and coding of two-dimensional crossplots speeds up data processing capabilities and improves computational efficiency.
(4) The introduction of OPTN into the crossplot not only solves the problem of data points overlap, but also involves only the calculation of the OPTN adjacency matrix, which is easy to process and implement by computer. In addition, the crossplot partition is essentially a symbolization process, which makes the method robust to noise.

Although the proposed method shows valuable results, there are still some issues that need further research. Firstly, the number of network nodes varies with the size of the ruler. This causes the transition entropy, which measures the transition regularity between nodes, to be a relative indicator when analysing crossplots with definite coupling state. It changes with the value of the ruler. The extraction of suitable parameters from the OPTN as an evaluation indicator deserves further study. Secondly, the characteristics and conclusions of the proposed method are all based on the crossplots constructed by the same type of time series, while crossplots constructed from different types of coupled time series have not been involved. Thirdly, the rationality of the phase radial partition method is based on the comparison of examples, without systematic theoretical proof. Fourthly, only a single coupled model is analysed, and the analysis of multiple coupled models are not compared. Investigation of other coupled models may improve the comprehensiveness. In the following research work, these issues are taken as the research object for further in-depth research.

## 5. Conclusion

In this study, by introducing OPTN into the crossplot, a new method to measure the coupling strength of bivariate time series was proposed. The analysis results on unidirectional coupled Lorenz model showed that the method was not only overcoming the problem of data points overlap inherent in crossplot, but also had the advantages of being less affected by parameter settings, calculation efficiency, robustness, consistency with theoretical values and ideal for short time series. The actual EEG dataset analysis results showed that the new method was also effective for weak signals with synchronous relationships. The method and the complex network formed by the method can also be further used for physiological state assessment, myoelectric decoding, information and financial fields where information synchronous relationship exist (the Matlab program code and results related to CPTE algorithms can be downloaded from this link: https://doi.org/10.5061/dryad.z34tmpgkd).

## Data Availability

The raw data supporting the conclusions of this article will be made available by the authors, without undue reservation, to any qualified researcher. Data are available from the Dryad Digital Repository: https://doi.org/10.5061/dryad.z34tmpgkd [53]. The data are provided in electronic supplementary material [54].
